# Letter from the Editor in Chief

**DOI:** 10.19102/icrm.2025.16095

**Published:** 2025-09-15

**Authors:** Devi Nair


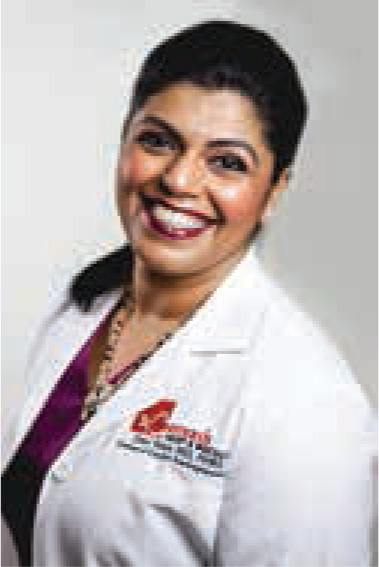


Dear Colleagues,

September was a month of both scientific progress and collective reflection. The ESC Congress 2025 once again showcased how rapidly cardiac rhythm management is evolving—from transformative ablation energy sources to physiologic pacing and digital integration. This issue of *The Journal of Innovations in Cardiac Rhythm Management* captures that same spirit of innovation through clinical insight, technical mastery, and a deep focus on patients with challenging arrhythmias.

## Advances in Science and Clinical Practice

This month’s manuscripts highlight how creativity and precision continue to drive the field forward:

- ***Epicardial ablation for inappropriate sinus tachycardia.*** Inappropriate sinus tachycardia remains one of the most difficult-to-treat arrhythmias, often refractory to medical therapy and standard ablation. Eldesouky and colleagues^1^ describe a hybrid surgical–electrophysiological case in which epicardial ablation achieved sinus node modification after endocardial ablation proved unsafe due to phrenic nerve proximity. This elegant approach exemplifies innovation applied to a persistently challenging clinical problem.
- ***Marshall bundle alcohol ablation.*** Afzal and collaborators^2^ provide a detailed, step-by-step guide to ethanol infusion in the vein of Marshall—a technique now re-emerging as a valuable adjunct to pulmonary vein isolation for persistent atrial fibrillation. Their practical discussion demystifies a complex procedure and illustrates how experience and preparation can expand the success of durable ablation strategies.
- ***Cardiac resynchronization therapy defibrillator implantation via the vein of Marshall.*** Karimli and colleagues^3^ present an ingenious solution for a rare anatomical obstacle—coronary sinus ostial atresia—by successfully implanting a left ventricular lead through the vein of Marshall. The case underscores the importance of anatomical understanding and procedural adaptability in cardiac resynchronization therapy.
- ***“Brugadaphobia” and the challenge of risk perception.*** Littmann and Bock^4^ explore the psychological dimension of electrophysiology through a striking case of patient-driven implantable cardioverter-defibrillator implantation in a low-risk Brugada phenocopy case. Their discussion of “brugadaphobia” highlights the modern clinician’s role in balancing data-driven reassurance with patient fears shaped by information overload.

## Spotlight: ESC Congress 2025

This year’s ESC Congress reinforced how innovation is reshaping rhythm management practice:

- ***Pulsed field ablation.*** Multicenter data demonstrate durable pulmonary vein isolation with a superior safety profile, minimizing risks of collateral injury to surrounding structures.
- ***Conduction system pacing.*** The late-breaking PhysioSync-HF trial, presented by André Zimerman, compared conduction system pacing (CSP) with biventricular pacing in patients with heart failure and left bundle branch block. Contrary to earlier expectations, CSP was inferior to biventricular pacing for the composite endpoint of death, heart failure hospitalization, urgent visits for heart failure, and left ventricular ejection fraction improvement at 12 months. This pivotal study tempers prior enthusiasm for CSP as a universal alternative, underscoring that physiologic pacing must be individualized based on anatomy, disease substrate, and experience.
- ***Atrial fibrillation ablation.*** Trials such as PROMPT-AF highlight the benefit of adjunctive approaches—like Marshall bundle ethanol infusion—in improving rhythm durability in persistent AF.
- ***Digital and artificial intelligence integration.*** Artificial intelligence–based algorithms and remote monitoring platforms are demonstrating growing value in arrhythmia prediction, workflow efficiency, and personalized care.

Collectively, these findings reflect a maturing field where bold innovations are increasingly tested through rigorous, outcome-driven trials.

## Looking Ahead

From tackling refractory conditions like inappropriate sinus tachycardia to refining ablation and pacing strategies, September’s issue mirrors the forward motion seen on the global stage at ESC. Each contribution in this edition reinforces the guiding principle of our field: that progress arises from thoughtful experimentation, collaboration, and an unrelenting focus on improving patient outcomes.

We hope this issue provides both inspiration and practical insight as you continue your work advancing heart rhythm care.

Warm regards,


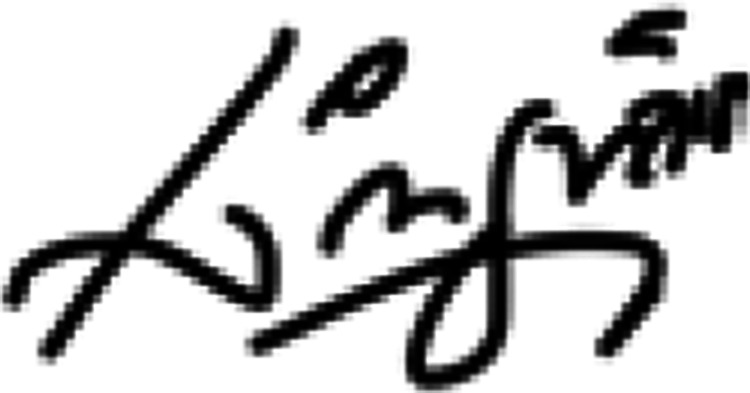


Dr. Devi Nair, md, facc, fhrs

Editor-in-Chief

*The Journal of Innovations in Cardiac Rhythm Management*

Director of the Cardiac Electrophysiology & Research,

St. Bernard’s Heart & Vascular Center, Jonesboro, AR, USA

White River Medical Center, Batesville, AR, USA

President/CEO, Arrhythmia Research Group

Clinical Adjunct Professor, University of Arkansas for Medical Sciences

Governor, Arkansas Chapter of the American College of Cardiology

drdgnair@gmail.com
